# Microclusters as T Cell Signaling Hubs: Structure, Kinetics, and Regulation

**DOI:** 10.3389/fcell.2020.608530

**Published:** 2021-01-26

**Authors:** Lakshmi Balagopalan, Kumarkrishna Raychaudhuri, Lawrence E. Samelson

**Affiliations:** Laboratory of Cellular and Molecular Biology, Center for Cancer Research, National Cancer Institute, National Institutes of Health, Bethesda, MD, United States

**Keywords:** TIRF-SIM, lattice light sheet microscopy, vesicle traffic, microclusters, live cell imaging

## Abstract

When T cell receptors (TCRs) engage with stimulatory ligands, one of the first microscopically visible events is the formation of microclusters at the site of T cell activation. Since the discovery of these structures almost 20 years ago, they have been studied extensively in live cells using confocal and total internal reflection fluorescence (TIRF) microscopy. However, due to limits in image resolution and acquisition speed, the spatial relationships of signaling components within microclusters, the kinetics of their assembly and disassembly, and the role of vesicular trafficking in microcluster formation and maintenance were not finely characterized. In this review, we will summarize how new microscopy techniques have revealed novel insights into the assembly of these structures. The sub-diffraction organization of microclusters as well as the finely dissected kinetics of recruitment and disassociation of molecules from microclusters will be discussed. The role of cell surface molecules in microcluster formation and the kinetics of molecular recruitment via intracellular vesicular trafficking to microclusters is described. Finally, the role of post-translational modifications such as ubiquitination in the downregulation of cell surface signaling molecules is also discussed. These results will be related to the role of these structures and processes in T cell activation.

## Introduction

The central event in the initiation of the adaptive immune response to foreign antigen is the interaction of the T cell antigen receptor (TCR) with an antigenic peptide presented by a protein encoded by the major histocompatibility complex (pMHC). The rapid biochemical events that then transpire, defined as T cell activation, have been the subject of extensive research for over three decades. Rapid recruitment and activation of Src family protein tyrosine kinases (PTK) and ZAP-70 lead to phosphorylation of tyrosine residues on the cytosolic regions of the TCR (the CD3 and TCRζ chains), adapter proteins (LAT and SLP-76), and various enzymes (Itk and PLC-γ1). These phosphorylations, in turn, lead to creation of sites for SH2 domain-mediated binding, leading to formation of protein complexes and to the activation of many of the bound enzymes (Weiss and Littman, 1994; Smith-Garvin et al., 2009; Balagopalan et al., 2010; Samelson, 2011; Courtney et al., 2018). These multiple events occur in the first seconds to minutes following TCR-pMHC engagement. Subsequently and dependent on these proximal events, further phosphorylations (primarily due to activation of protein serine kinases) and other enzymatic events lead to activation of transcription and to global cellular changes mediated by cytoskeletal reorganization. The most dramatic example of the latter is the generation of the immune synapse (IS) that forms between the T cell and an antigen-presenting cell (APC). The initial description of the IS was that of a segregated bulls-eye structure with a centralized TCR (the cSMAC) surrounded by the integrin LFA-1 (the pSMAC), with large molecules such as the phosphatatase CD45 excluded from the central region (Monks et al., 1998; Grakoui et al., 1999). IS formation was observed to take place about 10–30 min after TCR stimulation, while the biochemical events described above occur in seconds, indicating that the IS does not trigger initial TCR signals (Lee et al., 2002).

An early consequence of TCR-pMHC binding is the aggregation of the TCR and many of the above-described signaling molecules in structures known as microclusters. Visualization of these submicron-sized bodies was enabled by using high-speed confocal microscopy (see Box 1 in Supplementary Material) to visualize T cell activation in live cells expressing fluorescently tagged signaling molecules. Microclusters are sites of T cell activation as evidenced by the accumulation of tyrosine-phosphorylated proteins (Bunnell et al., 2002). Extensive analysis revealed that a substantial number of signaling molecules defined biochemically to be involved in T cell activation (as described above) were found within microclusters. Our original studies were performed on cells activated by anti-TCR antibodies on glass, while several subsequent studies by others employed activation by pMHC conjugated to planar lipid bilayers on glass and total internal reflection fluorescence microscopy (TIRFM; see Box 1 in Supplementary Material) (Campi et al., 2005; Yokosuka et al., 2005). More recently, advanced imaging techniques such as lattice light sheet microscopy (LLSM; see Box 1 in Supplementary Material) have enabled the visualization of microclusters at the initiation of T cell contact, thus confirming the role of these structures as signaling units that drive T cell activation (Ritter et al., 2015).

In this minireview, we aim to summarize recent insights into the organization and formation of microclusters and discuss the regulation of these structures via endocytic and exocytic mechanisms. Along the way, we will highlight the new imaging methodologies that have enabled these novel insights.

## Spatial Organization of Microclusters

Since the discovery of microclusters, the spatial organization of signaling molecules in these structures has been extensively studied. In the initial description of microclusters, the exclusion of large glycoproteins, CD43 and the phosphatase CD45, from microclusters, similar to their exclusion from the cSMAC of the IS, was described (Bunnell et al., 2002). More recently, the accumulation of LFA-1 surrounding the TCR microcluster to form an “adhesion ring” in microscale during the initiation of T cell activation was observed, reminiscent of the bulls-eye organization of the IS (Hashimoto-Tane et al., 2016). Though microclusters are thought of as T cell activation units, assemblies of receptor and signaling proteins can be detected in the membrane of resting T cells, suggesting that smaller preformed “nanoclusters” may pre-segregate into specialized membrane domains prior to TCR triggering (Lillemeier et al., 2006, 2010; Crites et al., 2014). Despite considerable ambiguity on spatial distribution, structural organization, and nomenclature of these structures, most investigators believe that upon TCR ligation, these “nanoclusters” undergo concatenation, remixing, and aggregation to form larger TCR microclusters (Lillemeier et al., 2010; Sherman et al., 2011; Hu et al., 2016).

Multiple super-resolution microscopy techniques, including single-molecule localization microscopy (SMLM; see Box 1 in Supplementary Material), have been developed that allow more detailed studies of the structure of signaling complexes. These investigations have revealed nanoscale organization of the TCR and other important components of the signal transduction pathway. Early studies using photo-activation localization microscopy (PALM; see Box 1 in Supplementary Material) showed that the TCR and LAT are clustered in both unactivated and activated cells and that the extent of clustering increased after TCR activation (Lillemeier et al., 2010; Sherman et al., 2011). Also, the TCR and LAT clusters tend to be segregated from each other, with some overlap at “hotspots” (Sherman et al., 2011). The two studies detected different sizes of clusters, with the Lillemeier study finding significantly larger clusters. These differences are likely due to differences in their analytical approach as described in Table 1 below.

**Table 1:** Fundamental differences in analytical approaches employed by Lillemeier et al. and Sherman et al. to analyze SMLM data.

**Parameters**	**Lillemeier et al**.	**Sherman et al**.	**Implication**
Statistical method for cluster analysis	Ripley's K-function analysis	Pair correlation function (PCF)	Smaller clusters are under-reported in Ripley's functions, which should be used mainly to report separation distances rather than cluster size. PCF shows results uniformly across all length scales and is more appropriate for detecting small-scale clusters.
Poisson model	Standard Poisson null model	Heterogeneous Poisson model	Effects of plasma membrane heterogeneity are considered in the heterogeneous Poisson process, while the standard Poisson model can report membrane ruffles as clusters.
Intensity-based thresholding	Yes	No	Many molecules are gated as background and small clusters may be removed from analysis.

Another study using STED showed that the clusters are smaller than STED resolution, in the range of 50–70 nm (Balagopalan et al., 2015). Despite the discrepancy in cluster size, these studies agreed that the TCR and LAT were found in clusters that increased in size with T cell activation. Since these early reports, there has been increasing interest in developing methods to analyze clustering. These range from stand-alone programs such as DBScan (Ester et al., 1996) to the use of machine learning (Williamson et al., 2020). Both the principles of SMLM and data analyses have been reviewed recently (Wu et al., 2020).

The organization of other molecules including Lck, ZAP-70, Grb2, and SLP-76 in microclusters has also been studied (Lillemeier et al., 2010; Purbhoo et al., 2010; Hsu and Baumgart, 2011; Sherman et al., 2011; Rossy et al., 2013; Neve-Oz et al., 2015). ZAP-70 kinase mixes uniformly with TCR but shows only partial mixing with LAT. LAT clusters recruited Grb2 regardless of size, indicating that even small nanoclusters contain phosphorylated LAT and participate in T cell activation. Interestingly, LAT and SLP-76 were reported to form nanostructures with LAT tending to be in the center and SLP-76 distributed on the outside (Sherman et al., 2011). Further investigation showed that this LAT-SLP nanostructure develops during the spreading process (Barr et al., 2016), suggesting that the nanostructure of signaling complexes is dynamic and changes with time. This research has also revealed the difficulty in analyzing the patterns of multiple proteins. Methods such as the bivariate PCF can evaluate the interactions of two molecules, but it is difficult to determine how larger numbers of proteins interact. One study, which extended the analysis to three proteins, demonstrated concentric arrangements of molecules at LAT clusters, with VAV1 and PLCγ1 near LAT at the center, while SLP-76 was found at the periphery and actin was seen surrounding the clusters (Sherman et al., 2016). The analysis also examined the recruitment of subsets of proteins to LAT clusters. SLP-76 recruitment was promoted by interactions with PLCγ1 and actin. However, both PLCγ1 and actin associations with LAT clusters were independent of SLP-76. At this time, good statistical methods are not available to determine the organization of multiple proteins within the signaling complexes.

Despite the high precision reported by localization algorithms, visualization of proteins within complex structures has been hampered by several issues, including the accurate determination of the actual location of single molecules and limitations in the alignment of multi-color images. The density of the label also affects the accuracy of the image (Patterson et al., 2010). Current SMLM techniques rarely give true counts of the number of molecules; both overcounting and undercounting errors are common (Krizek et al., 2011). In particular, SMLM methods tend to produce multiple localizations from the same molecule. The difficulty in properly assigning these localizations to the correct molecule or the grouping of localizations remains one of the most stubborn problems (Erdelyi et al., 2015). Without this crucial correction, it is impossible to perform a detailed molecular analysis of microclusters and the IS. A recent technique, madSTORM (see Box 1 in Supplementary Material), addressed some of these issues and allowed the visualization of multiple targets at high resolution in a single sample. This method was able to produce high-resolution images of samples containing up to 20 different proteins (Yi et al., 2016). However, even in this scenario, only fixed samples could be used; each final image required capturing thousands of frames, and the process required several days to gather all the data. For now, SMLM is not able to determine microcluster structure in live cells.

**Figure 1:**
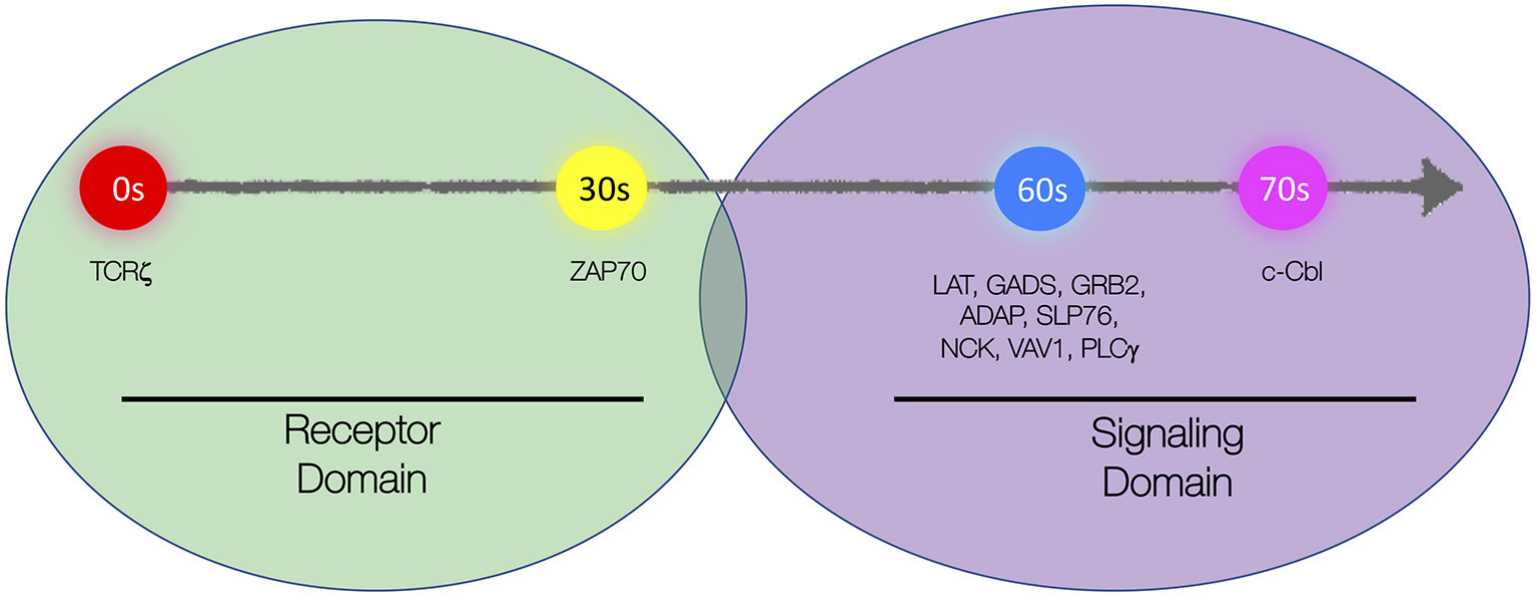
Schematic representation of spatial and kinetic organization of microclusters. Microclusters are organized into the “receptor domain” containing TCRζ and ZAP70 and the “signaling domain” containing several signaling proteins including LAT, GADS, GRB2, ADAP, SLP76, NCK, VAV, PLCγ1, and c-Cbl. Molecules are sequentially recruited to the microcluster with TCRζ being recruited first, ZAP70 recruited 30 s after TCRζ, LAT, and LAT-associated signaling proteins recruited simultaneously 30 s after ZAP70, and c-Cbl recruited 10 s after LAT.

More recent studies in live T cells using high-speed super-resolution microscopy techniques such as total internal reflection fluorescence structured illumination microscopy (TIRF-SIM; see Box 1 in Supplementary Material) have brought more clarity to the spatial organization TCR microclusters and their kinetics of assembly upon T cell activation (Yi et al., 2019). Two spatially segregated domains were identified within microclusters. TCR and ZAP-70 colocalized and marked the “receptor domain,” while LAT with its associated adaptor (GRB2, GADS, and SLP-76) and signaling proteins (ADAP, NCK, PLCγ, and VAV1) constituted the “signaling domain” of TCR microclusters. Sub-diffraction resolution images generated by TIRF-SIM showed that LAT was situated adjacent to the receptor domain proteins (TCRζ and ZAP-70) but did not colocalize with the latter. Likewise, adaptor and signaling proteins colocalized with each other and were positioned adjacent to and yet segregated from the receptor domain (Figure 1). The presence of such distinct domain organization within TCR microclusters might explain previously observed spatially segregated proteins islands of LAT and TCRζ at single-molecule resolution. Colocalization of TCRζ and ZAP-70 in the receptor domain echoes earlier observations of extensive mixing of ZAP-70 in TCRζ nanoclusters as originally observed by Sherman et al. With the discovery of such distinct domain organization of microclusters, questions arise on how the constituents of these two domains are brought in close proximity and if there are additional molecules (e.g., Shb, Lck, or others) that would be required to hold these complexes together (Welsh et al., 1998; Lindholm et al., 1999, 2002; Lo et al., 2018).

The idea of domains in the PM is not new. The lipid composition of the PM is not homogeneous, and it contains liquid-disordered and liquid-ordered domains. The liquid-ordered phase is enriched in cholesterol and sphingolipids and has long been studied as “lipid rafts,” where signaling proteins including TCR, Lck, and LAT segregate upon activation (Brdicka et al., 1998; Montixi et al., 1998; Zhang et al., 1998). Early evidence for the existence and functional relevance of “lipid rafts” came from resistance to detergent extraction, the effects of cholesterol depletion, and mutants that failed to localize to these domains (Munro, 2003). However, these methods do not identify these microdomains as they exist in the PM of cells. The visualization of lipid microdomains has been difficult because they are of a size below the resolution of conventional microscopy (Zacharias et al., 2002; Shaw, 2006). Phase-sensitive membrane probes and new imaging methodologies have allowed the direct visualization of membrane order in T cells. The IS has been shown to contain ordered membrane domains (Gaus et al., 2005; Owen et al., 2010). However, the presence of lipid order in microclusters is unclear. A study using FRET reported that several lipid raft markers do not accumulate in microclusters, suggesting that TCR microclusters form independently of lipid rafts (Hashimoto-Tane et al., 2010). However direct visualization of lipid order in activated T cells showed that the TCR resides in ordered plasma membrane domains that aggregate upon TCR engagement (Dinic et al., 2015). The role of lipid ordered domains in T cell signaling should still be considered in models of T cell activation (Courtney et al., 2018).

In addition to lipid-mediated phase separation, multivalent protein interactions lead to phase transitions within microclusters. LAT serves as an important scaffolding protein by virtue of its multiple interactions with other adapters and enzymes. Oligomerization of LAT mediated by multivalent interactions between LAT, LAT-bound adaptors, and adaptor-bound enzymes drives microcluster formation and has important functional outcomes (Houtman et al., 2006; Kortum et al., 2011; Coussens et al., 2013). *In vitro* reconstitution studies have demonstrated that LAT microclusters form due to a biophysical phase separation mediated by protein oligomerization (Su et al., 2016). Thus, both lipid and protein-mediated phase separation can create distinct physical compartments that facilitate signaling.

Most of the studies discussed above were performed using stimulatory surfaces such as antibody-coated cover glass or lipid bilayer systems. This raises the concern of whether such systems can accurately represent three-dimensional (3D) membrane dynamics that would naturally occur in a conjugate system of APC and T cell. With the advent of newer and sophisticated imaging techniques such as LLSM, some have started to capture the 3D membrane dynamics of T cell with unprecedented speed and resolution. There is growing evidence that indicates that dynamic membrane protrusions of T cells, called microvilli, play a critical role in T cell activation. High-resolution lattice lightsheet microscopy showed how microvilli play a crucial role in actively scanning the surface of APC for antigens (Cai et al., 2017). An approach using variable angle TIRF (VA-TIRF; see Box 1 in Supplementary Material) and super-resolution microscopy revealed the localization of fluorescently labeled TCR and signaling molecules nano-clustered at the tips of the microvilli (Jung et al., 2016; Ghosh et al., 2020). All these results generate a unified concept that preexisting nanoclusters or protein islands can be enriched in specialized membrane domains within dynamic microvilli. These nanoclusters can undergo intermixing and reorganization after TCR ligation thereby bringing receptor, adaptor, and signaling proteins into close proximity to generate intracellular signaling events.

## Microclusters are Assembled in Discrete Kinetic Steps

In contrast to the prediction of stochastic recruitment according to the “Protein Island” model, Yi *et al*. showed that individual proteins were recruited into the microcluster in a non-stochastic and stepwise sequential manner. Live-cell TIRF-SIM and TIRF microscopy approaches showed that, following TCR engagement, ZAP-70 was first recruited to TCR microclusters, followed by simultaneous recruitment of signaling and adaptor domain proteins (LAT, SLP-76, GRB2, ADAP, VAV1, NCK, and PLCγ). The simultaneous recruitment of LAT with its associated adaptors, signaling, and enzyme proteins is compatible with previous results, which established highly cooperative protein–protein interactions and stochastic cross-linking of multiprotein complexes (Houtman et al., 2006; Coussens et al., 2013). Recruitment of signaling domain proteins also leads to intracellular calcium flux, which indicates initiation of active signaling at the microclusters (Figure 1).

Distinct kinetic lags were established between recruitment of individual proteins in the microcluster. The assembly phase was followed by a disassembly or signal attenuation phase marked by recruitment of the E3 ubiquitin ligases, c-Cbl, which is involved in internalization and degradation of LAT signaling complexes (Balagopalan et al., 2007, 2011). LAT-bound signaling domain proteins showed a bimodal dissociation behavior from the microcluster. GRB2 and PLCγ showed slower dissociation kinetics, while GADS and SLP-76 showed rapid dissociation. Multiple mechanisms can be postulated for different kinetics of dissociation, such as inherently different affinities; different rates of dephosphorylation, ubiquitination, and endocytosis; and distinct pulling forces from the actin network on these structures (Barda-Saad et al., 2005; Yi et al., 2012; Kumari et al., 2015).

The kinetics of molecular recruitment to microclusters were shown to be sensitive to temperature and intracellular calcium levels. The kinetic lag between TCRζ and ZAP-70 showed a linear inverse relationship with temperature. However, the kinetic lag between ZAP-70 and GRB2 turned temperature-independent above 30°C. Because GRB2 recruitment depends on LAT clustering, the temperature independence of the ZAP-70 and GRB2 kinetic lag at this temperature could be attributed to the effect of temperature on membrane lipids, which can in turn influence LAT clustering (Tanimura et al., 2003; Su et al., 2016). Intracellular calcium was also found to have an impact on these kinetic lags, with calcium flux coincident with longer kinetic lags. Early microclusters that formed before calcium flux occurred showed negligible kinetic lags, and kinetic lags increased with time. A dose-dependent response in kinetic lags was observed by varying calcium concentration in the medium (Yi et al., 2019). This is consistent with an inhibitory role for calcium in the recruitment kinetics of proteins. In support of calcium flux dampening T cell signaling, our previous study reported that calcium chelation led to increased phosphorylation of signaling proteins and increased microcluster size (Balagopalan et al., 2018). Interestingly, an increase in intracellular calcium concentration led to reduced TCR mobility and promoted actin polymerization (Dushek et al., 2008), suggesting that calcium flux may regulate signaling protein kinetics via multiple pathways.

Advanced understanding of the kinetics of recruitment of molecules at the TCR microcluster have identified new “control nodes” in the kinetic proof-reading model of TCR signaling. According to this model, TCR signaling is a multi-step kinetic process in which progression to a subsequent step is contingent on achieving a “signaling threshold” or “signaling competent state” at the preceding kinetic step. Therefore, the duration (kinetic lag) and dissociation constant of each kinetic step is a major determinant of its progression to the next step (McKeithan, 1995; Lever et al., 2014). The kinetic lags observed by Yi *et al*. would directly feed into the kinetic parameters of a proof-reading model. Kinetic lags are also drastically altered after calcium flux. Therefore, TCR activation threshold and kinetic parameters of the proof-reading model will also be different before and after calcium flux. Calcium-dependent increases in kinetic lags can also act as a negative feedback mechanism to limit TCR signaling after a certain threshold. Kinetic lags between recruitment of signaling domain proteins and c-Cbl are also important. Recruitment of c-Cbl marks the dissociation of the signaling complexes in the microclusters and contributes to the window for active signaling at the microcluster. Thus, calcium-dependent stepwise assembly of microcluster components followed by bimodal dissociation of signaling proteins from microclusters represent new modes of T cell signal regulation.

## Regulation of Signaling from Microclusters Via Endocytosis and Recycling

Imaging of live T cells in real time during activation has revealed the changing signaling components and dynamics of microclusters. Soon after microclusters form, molecular mechanisms are activated to disassemble them and regulate the extent of signaling. These include recruitment of inhibitory receptors or adapters that either compete for binding with ligand or recruit phosphatases that allow for dephosphorylation of tyrosine residues and stochastic release of SH2 domain-containing proteins (Acuto et al., 2008; Yokosuka et al., 2010, 2012; Kong et al., 2019). Activation-induced protein endocytosis at microclusters is another effective way to regulate signaling duration by rapidly altering the subcellular locations of signaling proteins (Balagopalan et al., 2009). The recruitment of the E3 ubiquitin ligase c-Cbl coincides with microcluster disassembly and endocytosis of signaling molecules (Bunnell et al., 2002; Yokosuka et al., 2005; Yi et al., 2019). Dynamic disassembly of microcluster components indicates signal termination because reducing the dissociation of microclusters results in increased T cell signaling (Mossman et al., 2005; Barr et al., 2006; Balagopalan et al., 2007; Nguyen et al., 2008; Hashimoto-Tane et al., 2010; Lasserre et al., 2010; Vardhana et al., 2010).

Several studies have collectively shown that following T cell activation, increased receptor endocytosis, diminished recycling, and an increase in degradation causes a reduction in the number of TCR molecules at the plasma membrane (Alcover and Alarcon, 2000; Geisler, 2004). In a spatial context, signal initiation is thought to occur at the periphery of the IS and terminate at the cSMAC where TCRs are centrally accumulated and then internalized (Lee et al., 2003; Varma et al., 2006). A recent study using photoactivation to follow endocytosed TCR in real time reported that TCR endocytosis increased upon T cell stimulation and internalized TCR sorted into an endosomal compartment marked by flotillins that control recycling of TCR to the immunological synapse (Compeer et al., 2018). The strength of TCR signal plays a role in signal termination, with both weak and strong stimuli causing recruitment of signaling microclusters in the pSMAC and cSMAC, but strong ligands inducing TCR internalization from the cSMAC (Cemerski et al., 2008). Surprisingly, a study using Correlative *L*ight and Electron Microscopy (CLEM; see Box 1 in Supplementary Material) revealed that the majority of the centrally accumulated TCRs (in an IS formed on a lipid bilayer) are located on extracellular microvesicles (Choudhuri et al., 2014) that may serve as a channel for cell-to-cell communication with the APC (Mittelbrunn et al., 2011). Studies from our laboratory revealed that the dissipation of LAT and SLP-76 molecules away from early sites of microcluster formation are endocytic events (Barr et al., 2006; Balagopalan et al., 2007). While SLP-76 is endocytosed in a clathrin-independent mechanism, LAT is endocytosed via multiple pathways. After internalization from the PM, a portion of LAT undergoes retrograde trafficking to the Golgi (Carpier et al., 2018) and is delivered back to the synapse in an anterograde trafficking pathway regulated by golgin molecules (Zucchetti et al., 2019). Interestingly, in the case of both TCR and the adapters SLP-76 and LAT, internal pools of signaling-competent endosomes have been detected (Barr et al., 2006; Yudushkin and Vale, 2010; Evnouchidou et al., 2020), indicating that at least some of the endocytosed molecules are still active. Signals emanating from complexes located in endosomes might be qualitatively and/or quantitatively different from signals generated from complexes located at the plasma membrane.

**Figure 2:**
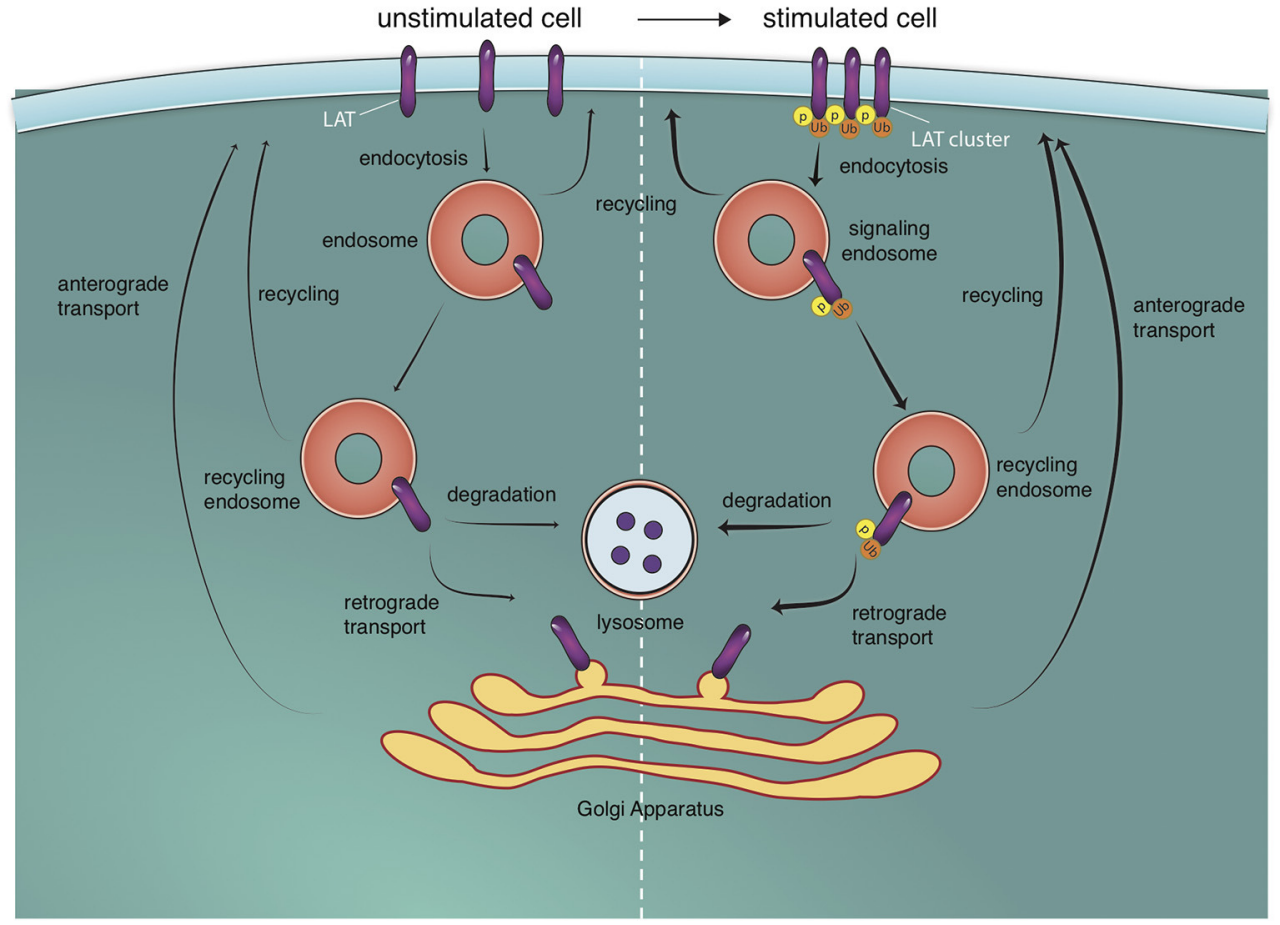
Endosomal trafficking pathways to and off the T cell surface. T cell stimulation triggers the formation of microclusters. LAT molecules (and other signaling molecules) in microclusters are phosphorylated (p) and ubiquitylated (u). These molecules are internalized into endosomes from which they can potentially signal. They can then proceed to the lysosome where they are degraded or be recycled back to the cell surface. Recycling back to the cell surface can occur directly or via retrograde trafficking to the golgi apparatus and anterograde trafficking from the golgi apparatus to the plasma membrane. These trafficking pathways also exist in unstimulated cells but are increased upon T cell activation (indicated by thicker arrows).

As indicated by the recruitment of the E3 ligase c-Cbl to microclusters, an important molecular mechanism that determines the cellular fate of endocytosed signaling molecules is ubiquitination. Inhibition of cellular ubiquitination increased microcluster lifetime and signal persistence (Barr et al., 2006; Vardhana et al., 2010). The Cbl family of ubiquitin ligases promote the ubiquitination and degradation of ZAP-70, Lck, LAT, SLP-76, Vav1, and WASP (Rao et al., 2000, 2002; Miura-Shimura et al., 2003; Barr et al., 2006; Balagopalan et al., 2007; Reicher et al., 2012). We have shown previously that the endocytosis of microclusters containing LAT and SLP-76 is regulated by c-Cbl mediated ubiquitination, and inhibition of c-Cbl function increases microcluster lifetime (Barr et al., 2006; Balagopalan et al., 2007). Ubiquitin is a sorting signal that regulates trafficking events within the endocytic pathway (Piper et al., 2014), and ubiquitin-binding ESCRT-I protein Tsg101 recognizes ubiquitinated chains of signaling proteins to be transported to lysosomes (Vardhana et al., 2010). Another important negative feedback mechanism is the phosphorylation of the adapters SLP-76 by the serine–threonine kinase HPK1 (hematopoietic progenitor kinase 1). Phosphorylation of SLP-76 on serine promotes 14-3-3 binding (Di Bartolo et al., 2007; Lasserre et al., 2011), resulting in SLP-76 ubiquitination and degradation (Wang et al., 2012). Thus, multiple endocytic feedback loops operate to regulate the extent of signaling from microclusters at the IS (Figure 2).

Consistent with a role for protein ubiquitination in signal termination, a LAT mutant that cannot be ubiquitinated (LAT 2KR) displayed enhanced signaling (Balagopalan et al., 2011; Kunii et al., 2013; Rodriguez-Pena et al., 2015). In a recent study, we examined the correlation between LAT ubiquitination and LAT cellular trafficking by comparing the cellular route of 2KR and wild-type LAT. Though internalization of LAT is Cbl and ubiquitin-dependent, ubiquitin-resistant 2KR LAT and wild-type LAT were internalized at comparable rates, indicating that LAT ubiquitination itself is not necessary for internalization of LAT (Balagopalan et al., 2020). LAT is predominantly monoubiquitinated (Balagopalan et al., 2011) and though a single Ub is perhaps an insufficient endocytic signal, the aggregate effect of multiple Ubs on multiple microcluster proteins may trigger endocytosis (Piper et al., 2014). Critically, LAT ubiquitination served as a signal for lysosomal trafficking and degradation, thus preventing LAT recycling to the cell surface. In 2KR LAT molecules that cannot be ubiquitinated, mutant LAT continues to recycle back to the cell surface, thus increasing the protein lifetime of LAT and providing a cellular trafficking correlate for the enhanced function of 2KR LAT (Balagopalan et al., 2020).

## Recruitment of Vesicles Containing Signaling Molecules to the IS

The IS is a site of bi-directional membrane trafficking. In addition to endocytic events described above, polarized traffic of exocytic vesicles to the IS is crucial for T cell function. During the formation of the IS, the movement of the microtubule organizing center (MTOC) toward the APC results in the directed secretion of cytokines in helper T cells (Kupfer et al., 1991) and secretory granules in cytotoxic T cells (Stinchcombe et al., 2006). Several recent studies have described the vesicular delivery of signaling molecules important in early activation of T cells. Signaling molecules critical for T cell activation, such as TCRζ, Lck, and LAT, are not present at the plasma membrane exclusively. They also reside in distinct, non-overlapping intracellular compartments (Soares et al., 2013) that are rapidly polarized toward the IS upon T cell activation (Ehrlich et al., 2002; Bonello et al., 2004; Das et al., 2004; Purbhoo et al., 2010). Signaling proteins in vesicular pools are delivered to the IS differentially via specific subpopulations of endocytic and exocytic machinery. Multiple regulatory proteins, such as Rab GTPases (Patino-Lopez et al., 2008; Carpier et al., 2018); t-SNAREs, SNAP-23, and syntaxin 4, and v-SNARES, VAMP-3, and VAMP-7 (Das et al., 2004; Larghi et al., 2013; Soares et al., 2013; Carpier et al., 2018); IFT system protein IFT20 (Finetti et al., 2009, 2015); Sorting Nexins (Osborne et al., 2015); and the ARP 2/3 activating WASH complex (Piotrowski et al., 2013), have been shown to play a significant role in the trafficking of TCRζ and proximal signaling proteins to the IS. The functional role of vesicular pools of signaling proteins were revealed in studies in which perturbations of the regulatory proteins involved in membrane trafficking interfered with T cell activation and function. Inhibition of SNARE-mediated fusion by tetanus toxin (Das et al., 2004), overexpression of dominant negative proteins (Patino-Lopez et al., 2008), and siRNA-mediated silencing of trafficking regulators (Finetti et al., 2009, 2015; Larghi et al., 2013; Soares et al., 2013; Carpier et al., 2018) have all clearly confirmed the importance of vesicular trafficking in T cell function. More recently, capture assays (Zucchetti et al., 2019) and optogenetic aggregation methods (Redpath et al., 2019) have emphasized the importance of the precise spatial organization of endocytic regulators in T cell activation.

**Figure 3:**
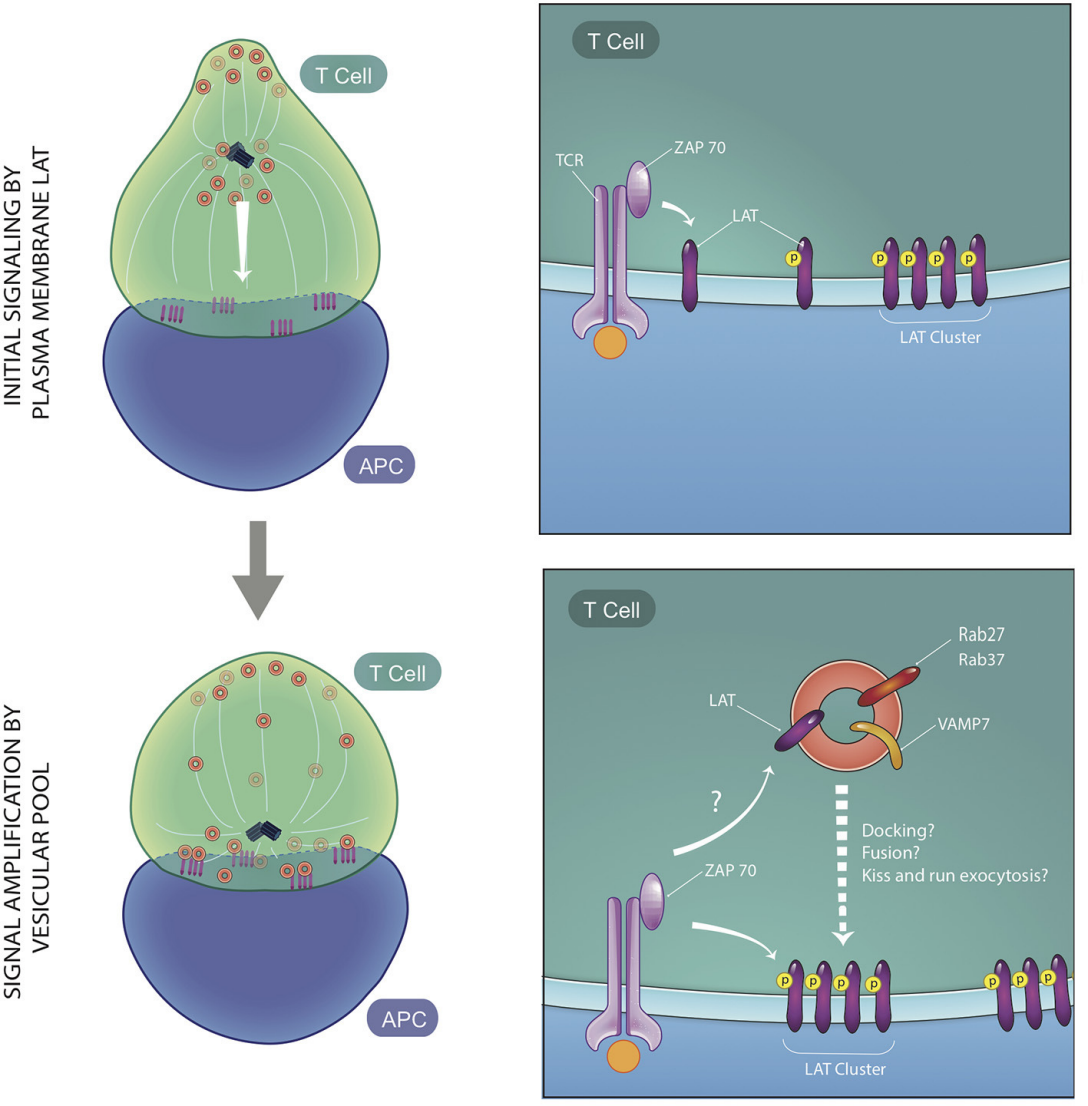
Two phases of early T cell activation. At early time points, vesicles containing signaling molecules are several microns away from the immune synapse, while plasma membrane-resident LAT is phosphorylated by ZAP70 and moves laterally to be recruited to microclusters. Soon after, vesicles expressing LAT, VAMP7, and Rabs are recruited to the immune synapse. These vesicles maintain and amplify signaling at the microclusters and interact dynamically with microclusters via either docking, fusion, or kiss and run exocytosis at microcluster sites. ZAP70 may trans-phosphorylate LAT on vesicles or cis-phosphorylate LAT at the PM, once vesicular fusion occurs (figure adapted from Balagopalan et al., *Nature Communications* 2018).

Though the critical role of vesicular traffic of signaling proteins in T cell activation has been clearly demonstrated, how and when the vesicular pools of signaling molecules regulate T cell activation remains less defined. The relative roles of vesicular vs. cell surface LAT pools for phosphorylation of LAT and TCR signal transduction has been controversial. While some studies proposed that plasma membrane LAT is the predominantly phosphorylated pool of LAT (Lillemeier et al., 2010; Sherman et al., 2011; Balagopalan et al., 2013), others proposed that docking or fusion of LAT vesicles at the IS is critical for LAT phosphorylation (Williamson et al., 2011; Larghi et al., 2013; Soares et al., 2013). Clues to the spatial and temporal contribution of vesicular signaling proteins came from fast 4D imaging using LLSM. LLSM enabled simultaneous imaging of surface and vesicular pools at the initiation of T cell activation, and revealed a role for both cellular pools. Early T cell activation was observed to occur in two phases: a first phase when recruitment of predominantly cell surface proteins formed microclusters, and a second phase, when the large pool of vesicles associated with the MTOC are recruited to the synapse (Figure 3) (Ritter et al., 2015; Balagopalan et al., 2018). In the second phase, directed movement of vesicles between microclusters on microtubules was observed. Vesicles displayed decreased speed and increased contact times at microclusters. Microclusters displayed fluorescence oscillations with an increase in fluorescence of LAT and signaling molecules coincident with when vesicles interacted with microclusters (Balagopalan et al., 2018). The observed oscillations indicate that vesicles sustain T cell signaling via delivery of a second wave of signaling molecules.

Once trafficked to the microcluster, LAT on vesicles could be either trans-phosphorylated by ZAP-70 localized at the PM or cis-phosphorylated once they fuse with the PM. There are contradictory reports about whether LAT vesicles undergo fusion with the PM. A flow cytometry approach to detect cell surface recruitment of LAT did not detect an accumulation of fused LAT at the PM (Larghi et al., 2013), leading to the conclusion that LAT vesicles dock close to but do not fuse with the PM. In contrast, interference with calcium-dependent vesicular fusion either by chelation of calcium or silencing of the calcium sensor synaptotagmin7 impeded microcluster formation, leading to a model in which calcium-dependent exocytosis of vesicles drives T cell signaling (Soares et al., 2013). Live-cell imaging using LLSM to directly visualize this process captured increases in LAT fluorescence when vesicles approached the IS. While the increases in fluorescence could be representative of vesicle fusion, a complete collapse of the vesicle was not observed (Balagopalan et al., 2018). This leads to the possibility that either vesicles dock transiently at microclusters or they undergo “kiss and run” exocytosis (Alabi and Tsien, 2013). It should be noted that the temporal acquisition speed of LLSM (4 s/frame) is too slow to allow for capture of exocytic events that occur very rapidly. A decrease in LAT signal from the vesicle after the flare is indicative of vesicular LAT delivery to the PM. In addition, no detectable LAT phosphorylation in subcortical vesicles was observed (Purbhoo et al., 2010), lending support to the model that LAT vesicles fuse with the synaptic membrane where LAT phosphorylation occurs (Figure 3). Polarized vesicle transport may also regulate the lipid composition at microclusters. A study of lipid order of sub-synaptic vesicles showed that they are not a homogenous population and vesicles display different degrees of membrane lipid order. Interestingly, LAT segregates into higher membrane order vesicles as it does on the PM (Ashdown et al., 2018). Thus lipid order-based sorting and delivery of cargo could contribute to maintaining lipid composition in the microcluster vicinity (Gagnon et al., 2012; Dinic et al., 2015).

Vesicle movement at the IS appears to be demarcated by microcluster location (Purbhoo et al., 2010; Balagopalan et al., 2018). Given the organization of the TCR and LAT in adjacent spatial domains, vesicular trafficking could be directed precisely to distinct nanoterritories at the IS. Spatial confinement of exocytosis to specialized plasma membrane regions has been reported in several biological systems (Yuan et al., 2015), and an important next step will be to investigate whether localized docking and/or exocytosis of vesicles occur at microcluster “hotspots.” Vesicle docking and fusion machinery such as SNARES (Chang et al., 2017) and exocyst components (Saez et al., 2019) may serve to mark microclusters as active docking or fusion zones. Microdomains enriched in intracellular calcium (Wei et al., 2009) could locally target calcium-dependent vesicle fusion. Just as differential usage of membrane trafficking regulators enables orchestration of endosome trafficking, defined spatial organization of fusion molecules could allow for targeting of distinct signaling molecules to discrete adjacent plasma membrane territories. Precise localization of fusion machinery and visualization of their accumulation kinetics are important next steps in uncovering the highly synchronized process of exocytosis and endocytosis at the IS.

## Future Goals

Increases in spatial and kinetic resolution in imaging technologies will certainly allow for novel insights into the interplay between the recruitment of molecules to the IS, compartmentalization of signaling components, vesicle movement, and location of signaling activity. The ability to combine super-resolution microscopy with readouts of function could provide insights into how signaling molecule organization at the nanoscale correlates with T cell activation and immune function. Multiplexing of biophysical measurements, high-throughput readouts and super-resolution imaging will be powerful next steps in uncovering novel insights to further understand immune cell signaling at the nanoscale. Such advances could potentially be used to manipulate T cell function in future immunotherapy.

## Author Contributions

LB and LS conceptualized, wrote, and edited the review, KR wrote sections of the review. All authors contributed to the article and approved the submitted version.

## Conflict of Interest

The authors declare that the research was conducted in the absence of any commercial or financial relationships that could be construed as a potential conflict of interest.
