# Supplementary material for: Microclusters as T Cell Signaling Hubs: Structure, Kinetics, and Regulation
Source: Front Cell Dev Biol. 2021 Jan 26;8:608530. doi: 10.3389/fcell.2020.608530 (PMC7870797; doi:10.3389/fcell.2020.608530)
Supplement: Supplementary file 1 [file Table_1.DOCX]

**BOX 1: Microscopy techniques used to study microclusters, microvilli and the IS of T cells**

**Confocal microscopy** is an optical imaging method that uses a physical pinhole to prevent out-of-focus light from reaching the detector. This improves the contrast in the final image resulting in a better picture than standard epifluorescence (Jonkman et al., 2020). Confocal microscopy is a diffraction limited technique with a resolution of 250 nm in xy and 700 nm in z. High speed confocal microscopy was used by (Bunnell et al., 2002) for the first description of microclusters in live T cells.

**Total Internal Reflection Microscopy (TIRFM)** allows imaging of only those fluorescent molecules that are located within a few hundred nm of the bottom coverslip (Crites et al., 2012). This is achieved by exciting the fluorophores with an evanescent wave rather than direct illumination with a light source. The evanescent wave is created by decreasing the incident angle of the excitation light beam until it is completely reflected by the cover slip. This technique is particularly useful due to low background and high signal to noise ratio and is well-suited for live cell imaging with high temporal resolution. First descriptions of microclusters on a lipid bilayer used TIRFM (Yokosuka et al., 2005) and it has been used in many studies since then.

**Lattice Light Sheet Microscopy** allows for gentle and fast imaging 4D imaging by producing a sheet of light for illumination (Chen et al., 2014). The z resolution is better than confocal microscopy. LLSM was used by (Ritter et al., 2015) to visualize actin dynamics at the cytotoxic IS, by (Cai et al., 2017) to look at T cell microvilli dynamics and by (Balagopalan et al., 2018) to visualize both PM and vesicle resident LAT simultaneously.

**Single Molecule Localization Microscopy (SMLM) techniques** provide the highest spatial resolution (less than 50 nm in XY and less than 100 nm in Z) by calculating the most likely location of a single fluorophore. Sparse single fluorophores are activated before imaging and then bleached before the next round of activation. Many activation and bleaching cycles are needed to produce a complete image. Photoswitchable fluorescent proteins are the fluorophores used in **Photoactivated Localization Microscopy (PALM)** and conventional antibodies and switchable dyes are used for **Stochastic Optical Reconstitution Microscopy (STORM)**. (Barr et al., 2017). (Lillemeier et al., 2010) and (Sherman et al., 2011) used PALM to look at TCR and LAT microclusters at the plasma membrane (PM) and (Williamson et al., 2011) used PALM to look at LAT clusters at the PM and in intracellular vesicles. **Multiplexed antibody size-limited direct Stochastic Optical Reconstitution Microscopy (madSTORM)** is a technique developed by (Yi et al., 2016) to allow targeting of multiple proteins using sequential binding and elution of antibodies. Time consuming image acquisition has led to SMLM usually being carried out in fixed samples.

**Stimulated Emission Depletion Microscopy (STED)** decreases the area of excitation by the selective deactivation of fluorophores on the periphery of the excitation beam (Hell and Wichmann, 1994). This minimizes the illumination area, thus increasing the resolution in the image. (Balagopalan et al., 2015)used STED to examine the size of microclusters.

**TIRF-Structured Illumination Microscopy (TIRF-SIM)** illuminates the sample with spatially structured light and the resulting super resolution image has twofold better resolution than a widefield image (100nm resolution). In combination with TIRF microscopy, it is well-suited for live cell imaging with high temporal resolution, but photobleaching is a drawback. (Murugesan et al., 2016) used this technique to visualize actin architecture at the IS and (Yi et al., 2019) used this technique to discover receptor and signaling domains within microclusters.

**Variable Angle-TIRFM (VA-TIRFM)** involves the recording of a stack of TIRF images, by gradually increasing the incident angle of the light beam on the sample. It allows for axial resolution in z of typically 10–20 nm and allows for mapping of structures in z. This technique in combination with SMLM was used to map TCRs and signaling molecules in relation to microvilli tips (Ghosh et al., 2020; Jung et al., 2016).

**Correlative Light and Electron Microscopy (CLEM)** combines fluorescence microscopy and electron microscopy thus allowing “lighting up” of cellular ultrastructure. (Choudhuri et al., 2014) used it to show microvesicles at the IS. Recent developments in probes, sample preparation, super-resolution fluorescence microscopy has resulted in resolution that is better matched between the two modalities.

**REFERENCES:**

Balagopalan, L., R.L. Kortum, N.P. Coussens, V.A. Barr, and L.E. Samelson. 2015. The linker for activation of T cells (LAT) signaling hub: from signaling complexes to microclusters. *J Biol Chem* 290:26422-26429.

Balagopalan, L., J. Yi, T. Nguyen, K.M. McIntire, A.S. Harned, K. Narayan, and L.E. Samelson. 2018. Plasma membrane LAT activation precedes vesicular recruitment defining two phases of early T-cell activation. *Nat Commun* 9:2013.

Barr, V.A., J. Yi, and L.E. Samelson. 2017. Super-resolution Analysis of TCR-Dependent Signaling: Single-Molecule Localization Microscopy. *Methods Mol Biol* 1584:183-206.

Bunnell, S.C., D.I. Hong, J.R. Kardon, T. Yamazaki, C.J. McGlade, V.A. Barr, and L.E. Samelson. 2002. T cell receptor ligation induces the formation of dynamically regulated signaling assemblies. *J. Cell Biol.* 158:1263-1275.

Cai, E., K. Marchuk, P. Beemiller, C. Beppler, M.G. Rubashkin, V.M. Weaver, A. Gerard, T.L. Liu, B.C. Chen, E. Betzig, F. Bartumeus, and M.F. Krummel. 2017. Visualizing dynamic microvillar search and stabilization during ligand detection by T cells. *Science* 356:

Chen, B.C., W.R. Legant, K. Wang, L. Shao, D.E. Milkie, M.W. Davidson, C. Janetopoulos, X.S. Wu, J.A. Hammer, 3rd, Z. Liu, B.P. English, Y. Mimori-Kiyosue, D.P. Romero, A.T. Ritter, J. Lippincott-Schwartz, L. Fritz-Laylin, R.D. Mullins, D.M. Mitchell, J.N. Bembenek, A.C. Reymann, R. Bohme, S.W. Grill, J.T. Wang, G. Seydoux, U.S. Tulu, D.P. Kiehart, and E. Betzig. 2014. Lattice light-sheet microscopy: imaging molecules to embryos at high spatiotemporal resolution. *Science* 346:1257998.

Choudhuri, K., J. Llodra, E.W. Roth, J. Tsai, S. Gordo, K.W. Wucherpfennig, L.C. Kam, D.L. Stokes, and M.L. Dustin. 2014. Polarized release of T-cell-receptor-enriched microvesicles at the immunological synapse. *Nature* 507:118-123.

Crites, T.J., L. Chen, and R. Varma. 2012. A TIRF microscopy technique for real-time, simultaneous imaging of the TCR and its associated signaling proteins. *J Vis Exp*

Ghosh, S., V. Di Bartolo, L. Tubul, E. Shimoni, E. Kartvelishvily, T. Dadosh, S.W. Feigelson, R. Alon, A. Alcover, and G. Haran. 2020. ERM-Dependent Assembly of T Cell Receptor Signaling and Co-stimulatory Molecules on Microvilli prior to Activation. *Cell Rep* 30:3434-3447 e3436.

Hell, S.W., and J. Wichmann. 1994. Breaking the diffraction resolution limit by stimulated emission: stimulated-emission-depletion fluorescence microscopy. *Opt Lett* 19:780-782.

Jonkman, J., C.M. Brown, G.D. Wright, K.I. Anderson, and A.J. North. 2020. Guidance for quantitative confocal microscopy. *Nat Protoc*

Jung, Y., I. Riven, S.W. Feigelson, E. Kartvelishvily, K. Tohya, M. Miyasaka, R. Alon, and G. Haran. 2016. Three-dimensional localization of T-cell receptors in relation to microvilli using a combination of superresolution microscopies. *Proc Natl Acad Sci U S A* 113:E5916-E5924.

Lillemeier, B.F., M.A. Mortelmaier, M.B. Forstner, J.B. Huppa, J.T. Groves, and M.M. Davis. 2010. TCR and Lat are expressed on separate protein islands on T cell membranes and concatenate during activation. *Nat Immunol* 11:90-96.

Murugesan, S., J. Hong, J. Yi, D. Li, J.R. Beach, L. Shao, J. Meinhardt, G. Madison, X. Wu, E. Betzig, and J.A. Hammer. 2016. Formin-generated actomyosin arcs propel T cell receptor microcluster movement at the immune synapse. *J Cell Biol* 215:383-399.

Ritter, A.T., Y. Asano, J.C. Stinchcombe, N.M. Dieckmann, B.C. Chen, C. Gawden-Bone, S. van Engelenburg, W. Legant, L. Gao, M.W. Davidson, E. Betzig, J. Lippincott-Schwartz, and G.M. Griffiths. 2015. Actin depletion initiates events leading to granule secretion at the immunological synapse. *Immunity* 42:864-876.

Sherman, E., V. Barr, S. Manley, G. Patterson, L. Balagopalan, I. Akpan, C.K. Regan, R.K. Merrill, C.L. Sommers, J. Lippincott-Schwartz, and L.E. Samelson. 2011. Functional nanoscale organization of signaling molecules downstream of the T cell antigen receptor. *Immunity* 35:705-720.

Williamson, D.J., D.M. Owen, J. Rossy, A. Magenau, M. Wehrmann, J.J. Gooding, and K. Gaus. 2011. Pre-existing clusters of the adaptor Lat do not participate in early T cell signaling events. *Nat Immunol* 12:655-662.

Yi, J., L. Balagopalan, T. Nguyen, K.M. McIntire, and L.E. Samelson. 2019. TCR microclusters form spatially segregated domains and sequentially assemble in calcium-dependent kinetic steps. *Nat Commun* 10:277.

Yi, J., A. Manna, V.A. Barr, J. Hong, K.C. Neuman, and L.E. Samelson. 2016. madSTORM: a superresolution technique for large-scale multiplexing at single-molecule accuracy. *Mol Biol Cell* 27:3591-3600.

Yokosuka, T., K. Sakata-Sogawa, W. Kobayashi, M. Hiroshima, A. Hashimoto-Tane, M. Tokunaga, M.L. Dustin, and T. Saito. 2005. Newly generated T cell receptor microclusters initiate and sustain T cell activation by recruitment of Zap70 and SLP-76. *Nat Immunol* 6:1253-1262.
